# Current status of high-risk HPV infection and correlation with multiple infections in cervical lesions in Western Guangzhou

**DOI:** 10.3389/fmed.2024.1252073

**Published:** 2024-04-17

**Authors:** Jianqi Li, He Lai, Honglei Qin, Dongmei Zhou, Yang Zhao, Xiujie Sheng

**Affiliations:** ^1^Department of Obstetrics and Gynecology, Guangdong Provincial Key Laboratory of Major Obstetric Diseases, Guangdong Provincial Clinical Research Center for Obstetrics and Gynecology, Guangdong-Hong Kong-Macao Greater Bay Area Higher Education Joint Laboratory of Maternal-Fetal Medicine, The Third Affiliated Hospital of Guangzhou Medical University, Guangzhou, China; ^2^Department of Obstetrics and Gynecology, Guangdong Women and Children Hospital, Guangzhou, China; ^3^Laboratory for Gynecologic Oncology, The Third Affiliated Hospital of Guangzhou Medical University, Guangzhou, China

**Keywords:** high-risk HPV, cervical lesions, multiple infections, age, Canton (Guangzhou)

## Abstract

**Objective:**

This study aims to investigate the current status of multiple HPV infection and its association with cervical lesions in the western region of Guangzhou.

**Methods:**

A retrospective analysis of clinical data from cervical cancer screening patients was conducted. The patients were grouped based on HPV genotypes and cervical pathology results to explore the prevalence of high-risk HPV infection and its relationship with cervical lesions in the western region of Guangzhou. The study also analyzed the relationship between high-risk HPV infection and cervical lesions among different age groups.

**Results:**

A total of 13,060 patients were included in the study, with an overall infection rate of 18.46% (2,411/13,060). Among them, the infection rate of HPV genotype 16 was 14.14% (341/2,411), HPV genotype 18 was 5.23% (126/2,411), and other 12 high-risk HPV genotypes accounted for 71.96% (1,735/2,411). When comparing the incidence of HSIL+ (high-grade squamous intraepithelial lesion or worse) among different HPV genotypes, the results showed that the HPV 16 infection group (47.50%) had a higher incidence than the HPV 18 infection group (25.40%) and the other 12 high-risk HPV genotypes group (15.97%; *P* < 0.05). In the multiple infection groups, the pathogenicity rates were 63.64% (7/11) for the 16+18 HPV infection group, 42.97% (55/128) for the 16+other 12 high-risk HPV genotypes infection group, 26.79% (15/56) for the 18+other 12 high-risk HPV genotypes infection group, and 57.14% (8/14) for the 16+18+other 12 high-risk HPV genotypes infection group. These rates were significantly different compared to the single infection group (*P* <0.01). Although there was no statistically significant difference in the incidence of cervical cancer between the HPV 16 infection group and the HPV 18 infection group, both groups had a higher incidence compared to the group with other 12 high-risk HPV genotypes infection (*P* < 0.05). Further analysis suggests that the severity of cervical lesions is not associated with the number of high-risk HPV infections, i.e., the severity of cervical lesions is unrelated to multiple HPV infections but is instead related to the pathogenicity of the HPV genotypes. The infection rate and multiple HPV infection rate of women under 35 years old were higher than those of women aged 35 and above (20% vs. 17.1%; 2% vs. 1.3%; *P* < 0.05). Moreover, the pathogenicity rate of HSIL+ among high-risk HPV infection increased with age.

**Conclusions:**

In the western region of Guangzhou, the overall infection rate of high-risk HPV is 18.46%. The severity of cervical lesions is unrelated to multiple HPV infections. The fundamental reason is the distinct pathogenicity of different HPV genotypes. The HSIL+ pathogenicity rates, from high to low, are in sequence for HPV 16, HPV 18, and the other 12 HPV types.

## 1 Introduction

Cervical cancer (CC) is the second most common malignancy among women globally and one of the most prevalent gynecological cancers. According to global cancer data in 2018 (1), out of 18.1 million newly diagnosed cancer cases worldwide, 570,000 were cervical cancer patients, accounting for ~3.2% of all cases. Each year, around 311,000 patients die from cervical cancer, representing ~3.3% of all cancer-related deaths worldwide. These statistics demonstrate the significant threat cervical cancer poses to women's health globally. The etiology of cervical cancer has been a focal point of research among scholars both domestically and internationally. In 1974, German virologist Harold zur Hausen first discovered the Human Papillomavirus (HPV) and confirmed its close association with cervical cancer (2). The International Agency for Research on Cancer stated in 2005 that persistent HPV infection is a direct factor in the development of cervical cancer and precancerous lesions (3).

HPV comprises over 160 different genotypes, with more than 40 genotypes confirmed to be associated with genital tract infections and 13–15 genotypes closely related to cervical squamous epithelial lesions and cervical cancer (4, 5). Due to significant differences in the capsid proteins between different HPV genotypes, cross-protective antibodies cannot be generated in hosts following HPV infection. This inability contributes to the occurrence of multiple and recurrent high-risk HPV infections (6). However, there has been ongoing controversy in the academic community regarding whether multiple high-risk HPV infections exacerbate the severity of cervical lesions or increase the risk of carcinogenesis compared to single high-risk HPV infections (7–9). Some argue that multiple high-risk HPV infections are one of the causes of persistent HPV infection, thereby exacerbating the severity of cervical lesions and increasing the risk of cancer. Others believe that the pathogenicity of HPV genotypes is the key factor determining the progression of cervical lesions and the risk of developing cancer.

The development of cervical cancer from HPV infection is a relatively lengthy process that involves the progression of cervical epithelial neoplasia, which can be categorized into CIN I, CIN II, and CIN III. According to the two-tier classification proposed by the World Health Organization in 2014 (10), benign atypia, mild dysplasia, flat warts, and CIN I are classified as low-grade squamous intraepithelial lesions (LSIL), while moderate dysplasia, severe dysplasia, CIN II, CIN III, and squamous cell carcinoma *in situ* are classified as high-grade squamous intraepithelial lesions (HSIL). Therefore, regular and effective cervical cancer screening strategies, along with cervical cancer vaccines, have become crucial measures for cervical cancer prevention and control. The Papanicolaou smear staining method, proposed by American scientist Papanicolaou in 1943, initiated the use of cytology as an important means of cervical cancer screening. However, as cytological screening advanced, its limitations became apparent, including low sensitivity, high false-negative rates, and poor reproducibility. Subsequent studies revealed that ~70% of cervical cancer patients are infected with HPV genotype 16 or 18, with 50% associated with genotype 16 infection and 20% associated with genotype 18 infection (11). The detection of high-risk HPV received attention in the academic community. High-risk HPV testing gradually became a co-screening method along with cytology for cervical cancer primary screening in women aged 30 and above, after initially being used as a triage method for patients with ASCUS (atypical squamous cells of undetermined significance) based on cytology results. The ATHENA study (12) confirmed the high sensitivity of HPV testing and its low rate of missed diagnoses for high-grade cervical intraepithelial neoplasia, making it a superior primary screening method compared to other screening methods. As a result, the U.S. FDA approved the Cobas HPV testing method as a primary screening method for cervical cancer in 2014 (13). However, some scholars have raised concerns, suggesting that HPV testing used solely as a primary screening method may detect a large number of individuals with transient HPV infections and potentially lead to missed diagnoses in HPV-negative cervical cancer patients.

Previous epidemiological investigations of high-risk HPV infection in Guangzhou have primarily focused on the eastern region, reporting infection rates of 14.07% (14), 13.94% (15), and 21.66% (16) respectively. Despite large sample sizes, these studies lack data from western Guangzhou and fail to delve into the relationship between multiple infections and cervical lesions.

Being the sole national tertiary Grade A hospital in western Guangzhou, our institution serves the Liwan District and Fangcun area, which collectively represent approximately one-fourth of the urban population of Guangzhou. Consequently, the outcomes derived from opportunistic screening for cervical cancer conducted in our hospital inherently reflect the prevalence of high-risk HPV in the western region of Guangzhou. Thus, our research contributes to addressing this gap by providing additional insights into this geographical area.

This study retrospectively analyzed the distribution of high-risk HPV infections in the western region of Guangzhou and further explored the relationship between multiple high-risk HPV infections and the severity of cervical lesions and the risk of carcinogenesis, aiming to provide evidence for the clinical diagnosis and treatment of cervical lesions.

## 2 General information

### 2.1 Study population

Clinical data (age, HPV genotype, liquid-based cytology results) of patients who underwent opportunistic cervical cancer screening at the Third Affiliated Hospital of Guangzhou Medical University from January 1, 2014, to December 31, 2022, were retrospectively analyzed.

### 2.2 Inclusion and exclusion criteria

#### 2.2.1 Inclusion criteria

1. Combined screening using liquid-based cytology and HPV genotyping tests.
2. Slides reviewed by pathology experts from the Third Affiliated Hospital of Guangzhou Medical University, with final diagnoses of Negative for Intraepithelial Lesion or Malignancy (NILM), Atypical Squamous Cells of Undetermined Significance (ASCUS), Low-Grade Squamous Intraepithelial Lesion (LSIL), High-Grade Squamous Intraepithelial Lesion (HSIL), or cervical cancer.
3. HSIL patients underwent surgical treatment by specialized doctors (attending physicians or higher positions) at the hospital, and the postoperative specimens were pathologically diagnosed by experts from our hospital.

#### 2.2.2 Exclusion criteria

1. Patients with acute vaginal inflammation or those who used vaginal medications or engaged in sexual activity within the past 3 days.
2. Pregnant or lactating women.
3. Previous history of cervical surgery or hysterectomy.
4. Recent use of hormonal medications.

#### 2.2.3 Ethical review

This study has obtained ethical approval from the Ethics Committee of the Third Affiliated Hospital of Guangzhou Medical University and was conducted in accordance with ethical standards (No. 2023C070106).

### 2.3 Criteria for cervical cancer screening

#### 2.3.1 Cervical liquid-based cytology (TCT) testing

Samples were collected by gynecologists at our hospital and interpreted using the revised 2001 TBS (The Bethesda System) for cervical cytology (17). The results were classified as negative, including normal range and benign reactive cellular changes, or positive, including atypical squamous cells (ASC), ASC of undetermined significance (ASC-US), ASC cannot exclude high-grade squamous intraepithelial lesion (ASC-H), low-grade squamous intraepithelial lesion (LSIL), high-grade squamous intraepithelial lesion (HSIL), or squamous cell carcinoma (SCC).

#### 2.3.2 High-risk HPV DNA testing

HPV testing was performed using the COBAS HPV detection method, including genotypes 16 and 18, as well as 12 other high-risk HPV genotypes. The other 12 high-risk genotypes were not included in the multiple high-risk HPV infection or single high-risk HPV infection groups due to the inability to determine if they were caused by a single or multiple HPV genotypes.

#### 2.3.3 Colposcopy-directed punch biopsies (CDB)

Colposcopy-directed punch biopsies were performed by experienced gynecologists at our hospital. Indications for colposcopy examination included abnormal cervical cytology results (LSIL or higher, ASCUS with positive high-risk HPV), suspicious symptoms or signs, positive HPV 16 or 18, suspicious vulvar, vaginal, or cervical lesions, and to assess the cervical transformation zone in postmenopausal women. Biopsy specimens were fixed in 10% formalin, sent immediately for pathological examination, and immunohistochemical staining was performed if necessary.

### 2.4 Pathological interpretation of results

#### 2.4.1 Pathological diagnosis

Pathological results served as the final basis for determining cervical lesions. Standardized procedures, including fixation, dehydration, transparency, paraffin embedding, serial sectioning, and routine hematoxylin and eosin (HE) staining, were followed to prepare pathological tissue slides. Slides were first reviewed by senior resident physicians at our hospital and then double-checked and finalized by two deputy chief physicians or higher-ranking physicians. In case of disagreement, a third physician with a deputy chief physician or higher-ranking position made the final interpretation. The pathological diagnosis of cervical intraepithelial neoplasia and cervical cancer followed the diagnostic criteria outlined in the second edition of “Obstetrics and Gynecology.”

### 2.5 Statistical analysis

SPSS version 21.0 statistical software was used for data analysis. Normality and homogeneity of variance tests were conducted for continuous variables. Normally distributed data with homogeneous variances were analyzed using *t*-tests and presented as mean ± standard deviation (mean ± SD). Non-normally distributed data or data with heterogeneous variances were analyzed using nonparametric Wilcoxon rank-sum tests. Categorical data were analyzed using chi-square tests or Fisher's exact tests. A *P*-value of < 0.05 was considered statistically significant.

## 3 Results

### 3.1 General information

A total of 13,060 patients undergoing opportunistic cervical cancer screening were included in the study. Among them, there were 2,411 cases of high-risk HPV infection, resulting in a prevalence rate of 18.46%. Based on the specific HPV genotypes, there were 341 cases of HPV-16 infection, 126 cases of HPV-18 infection, and 1,735 cases of infection with other high-risk HPV genotypes. Additionally, there were 11 cases of HPV-16+18 co-infection, 128 cases of HPV-16+other high-risk HPV co-infection, 56 cases of HPV-18+other high-risk HPV co-infection, and 14 cases of HPV-16+18+other high-risk HPV co-infection. A total of 10,649 cases were negative for high-risk HPV infection. The pathological examination results showed 10,517 cases of Negative for Intraepithelial Lesion or Malignancy (NILM), 917 cases of inflammatory cytological changes or cervicitis, 385 cases of Atypical Squamous Cells of Undetermined Significance (ASCUS), 676 cases of Low-Grade Squamous Intraepithelial Lesion (LSIL), 465 cases of High-Grade Squamous Intraepithelial Lesion (HSIL), and 106 cases of cervical cancer, Table 1.

**Table 1:** Genotypes of high-risk HPV infections and final pathological results in opportunistic cervical cancer screening patients.

**HPV genotype**	**NILM**	**ASCUS**	**LSIL**	**HSIL**	**CC**	**Total**
16	73	40	66	125	37	341
18	45	14	35	14	18	126
Other 12	681	270	507	248	29	1,735
16+18	1	0	3	5	2	11
16+other 12	24	16	33	45	10	128
18+other 12	14	4	23	13	6	56
16+18+other 12	2	1	3	6	2	14
Negative	10,594	40	6	7	2	10,649
Total	11,434	385	676	465	106	13,060

16, HPV 16 genotype; 18, HPV 18 genotype; other12, HPV other12 genotype; 16+18, HPV 16 genotype+ HPV 18 genotype; 16+ other 12, HPV 16 genotype+ HPV other12 genotype; 18+ other 12, HPV 18 genotype+ HPV other12 genotype; 16+18+ other 12, HPV 16 genotype+ HPV 18 genotype+ HPV other12 genotype; Negative, HPV negative.

### 3.2 Relationship between cervical lesion severity and genotypes/number of high-risk HPV infections

The incidence of high-grade squamous intraepithelial lesions (HSIL+) in different groups based on HPV infection genotypes and combinations, including HPV-16, HPV-18, other high-risk HPV genotypes, HPV-16+18 co-infection, HPV-16+other high-risk HPV co-infection, HPV-18+other high-risk HPV co-infection, and HPV-16+18+other high-risk HPV co-infection, was 47.50% (162/341), 25.40% (32/126), 15.97% (277/1,735), 63.64% (7/11), 42.97% (55/128), 26.79% (15/56), and 57.14% (8/14), respectively. The differences in HSIL+ incidence among these groups were statistically significant (*P* < 0.01), Table 2.

**Table 2:** Incidence of HSIL and above cervical lesions in different high-risk HPV infection groups.

**HPV genotype**	**LSIL-**	**HSIL+**	**Total**	***χ^2^***	***P***
16	179	162	341	212.8781	< 0.01
18	94	32	126		
Other 12	1,458	277	1,735		
16+18	4	7	11		
16+other 12	73	55	128		
18+ other 12	41	15	56		
16+18+ other 12	6	8	14		
Total	1,855	556	2,411		

Further intergroup comparisons were conducted among the different groups as follows: the HSIL+ incidence in the HPV-16 infection group was higher than that in the HPV-18 infection group (47.50% vs. 25.40%), with a significant statistical difference (*P* < 0.01). The HSIL+ incidence in the HPV-16 infection group was higher than that in the other 12 high-risk HPV infection group (47.50% vs. 15.97%), with a significant statistical difference (*P* < 0.01). The HSIL+ incidence in the HPV-18 infection group was higher than that in the other 12 high-risk HPV infection group (25.40% vs. 15.97%), with a significant statistical difference (*P* < 0.01). The HSIL+ incidence in the HPV-16+18 co-infection group was higher than that in the HPV-18 infection group (63.64% vs. 25.40%), with statistical significance (*P* < 0.05). The HSIL+ incidence in the HPV-16+other high-risk HPV co-infection group was higher than that in the other 12 high-risk HPV infection group (42.97% vs. 15.97%), with a significant statistical difference (*P* < 0.01). The HSIL+ incidence in the HPV-18+other high-risk HPV co-infection group was higher than that in the other 12 high-risk HPV infection group (26.79% vs. 15.97%), with statistical significance (*P* < 0.05). The HSIL+ incidence in the HPV-16+18+other high-risk HPV co-infection group was higher than that in the HPV-18 infection group (57.14% vs. 25.40%), with statistical significance (*P* < 0.05).

In addition, when comparing the HPV-16+18 co-infection group to the HPV-16 infection group, the HPV-16+other high-risk HPV co-infection group to the HPV-16 infection group, the HPV-18+other high-risk HPV co-infection group to the HPV-18 infection group, and the HPV-16+18+other high-risk HPV co-infection group to the HPV-16+18 co-infection group, although there were differences in HSIL+ incidence between these pairwise comparisons, the differences were not statistically significant (*P* > 0.05), Table 3.

**Table 3:** Pairwise comparisons of HSIL+ incidence among different high-risk HPV infection groups.

**HPV genotype**	***χ^2^***	***P***
16 vs. 18	18.521	< 0.05
16 vs. other 12	170.037	< 0.05
18 vs. other 12	7.546	< 0.05
16+18 vs. 16	1.111	>0.05
16+18 vs. 18	5.508	< 0.05
16+ other 12 vs. 16	0.771	>0.05
16+ other 12 vs. 12	59.353	< 0.05
18+ other 12 vs. 18	0.039	>0.05
18+ other 12 vs. 12	4.655	< 0.05
16+18+ other 12 vs. 16+18	0.007	>0.05
16+18+ other 12 vs. 16	0.189	>0.05
16+18+ other 12 vs. 18	4.764	< 0.05

### 3.3 Comparison of the carcinogenicity between multiple high-risk HPV infections and single high-risk HPV infections

The incidence of cervical cancer in the groups of HPV-16 infection, HPV-18 infection, other 12 high-risk HPV infections, HPV-16+18 co-infection, HPV-16+other high-risk HPV co-infection, HPV-18+other high-risk HPV co-infection, and HPV-16+18+other high-risk HPV co-infection were 10.85% (37/341), 14.29% (18/126), 1.67% (29/1,735), 18.18% (2/11), 7.81% (10/128), 10.71% (6/56), and 0% (2/14), respectively. There were significant statistical differences in the incidence of cervical cancer among the groups (*P* < 0.01), Table 4.

**Table 4:** Incidence of cervical cancer among different high-risk HPV infection groups.

**HPV genotype**	**None CC**	**CC**	**Total**	***χ^2^***	***P***
16	304	37	341	112.857	< 0.01
18	108	18	126		
Other 12	1,706	29	1,735		
16+18	9	2	11		
16+other 12	118	10	128		
18+ other 12	50	6	56		
16+18+ other 12	12	2	14		
Total	2,307	104	2,411		

Further intergroup comparisons were conducted as follows: the HPV-16 infection group had a higher incidence of cervical cancer compared to the other 12 high-risk HPV infection group (10.85% vs. 1.67%), with a significant statistical difference (*P* < 0.01). The HPV-18 infection group had a higher incidence of cervical cancer compared to the other 12 high-risk HPV infection group (14.29% vs. 1.67%), with a significant statistical difference (*P* < 0.01). The HPV-16+other high-risk HPV co-infection group had a higher incidence of cervical cancer compared to the other 12 high-risk HPV infection group (7.81% vs. 1.67%), with a significant statistical difference (*P* < 0.01). The HPV-18+other high-risk HPV co-infection group had a higher incidence of cervical cancer compared to the other 12 high-risk HPV infection group (10.71% vs. 1.67%), with a significant statistical difference (*P* < 0.01).

In addition, when comparing the HPV-16 infection group to the HPV-18 infection group, the HPV-16+18 co-infection group to the HPV-16 infection group, the HPV-16+18 co-infection group to the HPV-18 infection group, the HPV-16+other high-risk HPV co-infection group to the HPV-16 infection group, the HPV-18+other high-risk HPV co-infection group to the HPV-18 infection group, and the HPV-16+18+other high-risk HPV co-infection group to the HPV-16+18 co-infection group, although there were differences in the incidence of cervical cancer between these pairwise comparisons, the differences were not statistically significant (*P* > 0.05).

### 3.4 Analysis of high-risk HPV infection and subsequent cervical lesions in different age groups

A total of 13,060 patients were included in the study, among whom 2,411 had high-risk HPV infection and 10,649 were high-risk HPV negative. The patients were further divided into six age groups: below 25, 25–34, 35–44, 45–54, 55–64, and over 65 years. The high-risk HPV infection status and subsequent cervical lesion conditions were analyzed in each age group, Tables 5, 6.

**Table 5:** High-risk HPV infection status across different age groups.

**HPV genotype**	** < 25 years**	**25–34 years**	**35–44 years**	**45–54 years**	**55–64 years**	**>65 years**	**Total**
Negative	422	4,365	3,415	1,785	490	172	10,649
16	13	150	94	65	16	3	341
18	5	53	41	17	7	3	126
Other 12	111	748	508	248	91	29	1,735
16+18	0	9	1	1	0	0	11
16+ other 12	12	55	27	18	11	5	128
18+ other 12	5	30	13	3	1	4	56
16+18+ other 12	4	5	3	0	2	0	14
Total	572	5,415	4,102	2,137	618	216	13,060

**Table 6:** Cervical lesion status among individuals with high-risk HPV infection across different age groups.

**Age group (year)**	**NILM**	**ASCUS**	**LSIL**	**HSIL**	**CC**	**Total**
< 25	72	16	44	14	1	147
25–34	375	140	322	186	15	1,038
35–44	216	112	205	137	27	697
45–54	123	48	69	86	34	360
55–64	39	22	24	21	20	126
>65	11	7	6	8	7	43
Total	840	345	670	452	104	2,411

Among the 2,411 patients with high-risk HPV infection, 556 individuals had pathological results of HSIL or above (referred to as HSIL+). The overall prevalence of HSIL+ among high-risk HPV-infected individuals was 23.1% (556/2,411). Moreover, the incidence of HSIL+ showed a progressive increase with age. The rates were 10.2% (15/147) for those below 25 years, 19.36% (201/1,038) for those aged 25–34 years, 23.33% (164/697) for those aged 35–44 years, 33.33% (120/360) for those aged 45–54 years, 32.53% (41/126) for those aged 55–64 years, and 34.88% (15/43) for those over 65 years (Table 6).

Among the 13,060 patients included in the study, there were 5,987 females below the age of 35. Among them, 1,200 individuals were found to have high-risk HPV infection, resulting in a high-risk HPV infection rate of 20%. Additionally, 120 individuals had multiple high-risk HPV infections, corresponding to a multiple high-risk HPV infection rate of 2%. Among the 7,073 females aged 35 and above, 1,211 individuals were identified with high-risk HPV infection, leading to a high-risk HPV infection rate of 17.1%. Furthermore, 89 individuals had multiple high-risk HPV infections, resulting in a multiple high-risk HPV infection rate of 1.3%. The high-risk HPV infection rate and multiple high-risk HPV infection rate were higher in females below the age of 35 compared to those aged 35 and above, with statistically significant differences (*P* < 0.05), Tables 7, 8.

**Table 7:** Comparison of high-risk HPV infection status between women aged below 35 and women aged 35 and above.

	** < 35 years**	**≥35 years**	***χ^2^***	***P***
Total	5,987	7,073	18.391	< 0.01
HPV infection	1,200	1,211		
Infection rate (%)	20.0%	17.1%		

**Table 8:** Comparison of multiple high-risk HPV infection status between women aged below 35 and women aged 35 and above.

	** < 35 years**	**≥35 years**	***χ^2^***	***P***
Total	5,987	7,073	11.460	< 0.01
Multiple HPV infection	120	89		
Infection rate (%)	2.0%	1.3%		

## 4 Discussion

Cervical squamous intraepithelial neoplasia and cervical cancer are closely associated with persistent infection of high-risk HPV. Currently, over 160 genotypes of HPV have been identified, with more than 40 genotypes related to genital tract infections and 13–15 genotypes closely associated with cervical squamous intraepithelial neoplasia and cervical cancer. Different genotypes of HPV exhibit significant genetic variations in the coding of their capsid proteins, which essentially do not produce cross-protective antibodies after infection. This is one of the important factors leading to multiple and recurrent high-risk HPV infections.

Our study focuses on the population of the western region of Guangzhou, which accounts for approximately one-fourth of the urban population of the city. Previous research has primarily concentrated on data from the eastern region of Guangzhou. Therefore, our study serves to complement the existing literature by addressing this geographical gap and providing a more comprehensive perspective. Our retrospective analysis reveals that the overall prevalence of high-risk HPV infection in the western region of Guangzhou (18.46%) aligns with previous studies conducted in the eastern region (14–16). However, it is lower than the rates reported in Shenzhen (23.2%) (18), Taiwan (29.4%) (19), and certain areas of Hubei Province (44.4%) (20), while higher than those in Shanxi Province (14.8%) (21) and the Nyingchi City of Tibet (13.0%) (22).

Currently, there is no unified conclusion in the academic community regarding whether multiple high-risk HPV infections result in increased severity of cervical lesions or an elevated risk of developing cancer compared to single high-risk HPV infection. Some researchers believe that multiple high-risk HPV infections are one of the causes of persistent HPV infection, further exacerbating the degree of cervical lesions and increasing the risk of cancer (23). On the other hand, some scholars argue that the severity of cervical lesions and the risk of cervical cancer are not related to the number of high-risk HPV infections but rather to the pathogenicity of the genotypes involved (24–31).

Considering the risks and consequences of high-grade squamous intraepithelial lesions (HSIL+) and their importance, this study focuses on the relationship between the genotypes and number of high-risk HPV infections and HSIL+. Among different high-risk HPV infection genotypes, it was found that the incidence of HSIL+ was higher in the HPV 16 infection group compared to the HPV 18 infection group and the other 12 HPV infection group (*P*-values were all < 0.01). Additionally, the incidence of HSIL+ in the HPV 18 infection group was higher than that in the other 12 HPV infection group (*P* < 0.01). This indicates that in terms of the risks associated with HSIL+ development, HPV 16 > HPV 18 > other 12 high-risk HPV genotypes. By further comparing the groups, such as the 16+18 HPV infection group vs. the 16 HPV infection group, the 16+other 12 HPV infection group vs. the 16 HPV infection group, the 18+other 12 HPV infection group vs. the 18 HPV infection group, and the 16+18+other 12 HPV infection group vs. the 16+18 HPV infection group, although the number of high-risk HPV infections differed between the groups, there was no statistically significant difference in the risk of causing HSIL+ lesions due to the fact that the groups shared the same highest-risk HPV genotypes. Thus, it can be inferred that the number of high-risk HPV infections is not related to the severity of cervical lesions; instead, the severity of cervical lesions is associated with the pathogenicity of the HPV genotypes. This inference is further supported by observations from the comparison of different groups containing the highest-risk HPV genotypes, such as the 16+18 HPV infection group vs. the 18 HPV infection group, the 16+other 12 HPV infection group vs. the other 12 HPV infection group, the 18+other 12 HPV infection group vs. the other 12 HPV infection group. The group containing HPV genotypes with higher pathogenicity had a correspondingly higher incidence of HSIL+, and the differences were statistically significant (*P* < 0.05). This further confirms the aforementioned inference. A study by Xiang et al. (32) also found, through retrospective analysis of clinical data from 8,460 patients, that the risk of HSIL caused by HPV 16 and 18 genotypes was significantly higher than other genotypes. Additionally, the incidence of CIN was significantly higher in individuals with single HPV 16 infection compared to those with single HPV 18 infection, with significant statistical differences. This conclusion aligns closely with our study, which found that the risk associated with the development of HSIL+ lesions follows the order of HPV 16 > HPV 18 > other 12 high-risk HPV genotypes. Other reports have indicated that CIN patients infected with HPV 16 or 18 are more likely to experience rapid progression of lesions in the short term (33). Therefore, in cervical cancer screening, it is crucial to focus on the pathogenicity of the HPV genotypes involved in HPV infections, especially in the case of HPV 16 and 18. The number of HPV subgenotype infections is not an indicator for predicting the progression or severity of CIN lesions.

Similarly, whether multiple hrHPV infections increase the risk of cervical cancer compared to single hrHPV infections has been a hot topic of research with ongoing debates in the academic community. Some scholars believe that after the occurrence of multiple hrHPV infections, the presence of different genotypes of HPV may exhibit a certain synergistic effect, making it difficult for the body to completely clear the infection, thus increasing the risk of cervical cancer. On the other hand, another viewpoint suggests that multiple HPV infections are mainly dominated by the manifestation of one subgenotype, such as HPV-16, and an increased number of infections does not necessarily indicate a higher risk of carcinogenesis. In this study, no statistically significant difference was found in the occurrence rates of cervical cancer between the HPV-16 infection group and the HPV-18 infection group (*P* > 0.05). However, both the HPV-16 infection group and the HPV-18 infection group had significantly higher cervical cancer occurrence rates compared to the other 12 HPV infection groups (*P* < 0.01). This indicates that in terms of carcinogenicity, both HPV-16 and HPV-18 are higher than the other 12 high-risk HPV genotypes, but there is no significant difference in their carcinogenicity when comparing HPV-16 and HPV-18. Furthermore, when comparing the 16+18 HPV infection group vs. the HPV-16 infection group, the 16+18 HPV infection group vs. the HPV-18 infection group, the 16+other 12 HPV infection group vs. the HPV-16 infection group, the 18+other 12 HPV infection group vs. the HPV-18 infection group, the 16+18+other 12 HPV infection group vs. the 16+18 HPV infection group, the 16+18+other 12 HPV infection group vs. the HPV-16 infection group, and the 16+18+other 12 HPV infection group vs. the HPV-18 infection group, it was observed that although there were different numbers of hrHPV infections between the two groups, an increased number of hrHPV infections did not lead to an increased risk of cervical cancer. Moreover, as the two groups contained the same highest-risk HPV genotypes, there was no statistically significant difference in their ability to cause cervical cancer. Therefore, it can be inferred that there is no relationship between the number of hrHPV infections and the risk of cervical cancer, and the risk of cervical cancer is associated with the carcinogenicity of HPV genotypes.

By comparing the 16+other 12 HPV infection group vs. the other 12 HPV infection group and the 18+other 12 HPV infection group vs. the other 12 HPV infection group, it was found that the two groups being compared contained different highest-risk HPV genotypes, and the group with higher-risk HPV genotypes also had a higher incidence of cervical cancer, with statistically significant differences (*P* < 0.05). This further confirms that there is no relationship between the number of hrHPV infections and the risk of cervical cancer, and the risk of cervical cancer is associated with the carcinogenicity of HPV genotypes, which is consistent with the monoclonal origin theory of cancer. The higher risk of HPV-16 and HPV-18 may also be related to their longer duration of infection and lower clearance rates. Richardson et al. (34) found in a 2-year follow-up study of 621 patients that HPV-16 infection had the longest duration, with an average duration of 18.3 months. In conclusion, the number of HPV subgenotype infections cannot be considered as a predictor of the risk of cervical cancer. Clinically, for patients with multiple high-risk HPV infections, more attention should be given to the individual carcinogenic risks of specific genotypes.

Previous literature reports have suggested that women under 30 years of age are more likely to have multiple HPV infections compared to those over 30 years old (35, 36). In our study, we found that the prevalence of high-risk HPV infections and multiple high-risk HPV infections was higher in women under 35 years of age compared to those over 35 years old (20% vs. 17.1%; 2% vs. 1.3%), and these differences were statistically significant (*P* < 0.05). The higher prevalence among younger women can be attributed to factors such as poor sexual habits, including frequent sexual activity and multiple sexual partners. Additionally, the development of genotype-specific HPV antibodies requires exposure to HPV through sexual activity. Women under 35 years of age are still in the process of developing immunity against HPV infections, and their immune systems need time to mature. In contrast, women over 35 years of age have already developed immune systems capable of combating various HPV infections. Further analysis of 2,411 patients with high-risk HPV infections showed an increasing trend in the incidence of high-grade squamous intraepithelial lesions (HSIL+) with age. We propose the following explanations: (1) Persistent infection with high-risk HPV is the direct cause of cervical cancer and precancerous lesions, and the development of HSIL and higher-grade lesions requires a prolonged process. Therefore, the increasing trend in the incidence of HSIL+ with age reflects this progression. (2) Although older women have more mature immune systems to fight against various HPV infections, their immune response gradually decreases with age, making it difficult to completely clear high-risk HPV infections, leading to persistent infection. In conclusion, women under 35 years of age are more prone to high-risk HPV infections and even multiple infections compared to women over 35 years of age. The incidence rate of HSIL+ shows a progressive increase with the age of the infected individuals.

According to data published on the website of the National Health Commission of the People's Republic of China, the national routine immunization report shows that the reported HPV vaccine doses administered in various regions of the country were 3.417 million, 6.753 million, and 12.279 million doses for the years 2018, 2019, and 2020, respectively. While the number of vaccine doses administered has been increasing annually, the vaccination rates in the population remain relatively low. Currently, HPV vaccination is not included in the national immunization program and is administered on a voluntary and self-funded basis. The commission is actively promoting the inclusion of HPV vaccination in local public welfare policies where conditions permit. Since Guangzhou only introduced HPV vaccines in 2016, initially offering only the bivalent vaccine, the percentage of women in Guangzhou receiving HPV vaccines was extremely low (specific data for Guangzhou has not been disclosed). Therefore, the number of HPV vaccine recipients in this study was < 0.5% of the total screening population. This inadequate representation makes it impossible to fully assess the impact of HPV vaccination on HPV infection rates and cervical lesions in this study. Consequently, the vaccination status regarding HPV vaccines was not included in this research, representing a significant limitation and regrettable aspect of this study.

## Data availability statement

The original contributions presented in the study are included in the article/supplementary material, further inquiries can be directed to the corresponding author.

## Ethics statement

This study has obtained ethical approval from the Ethics Committee of the Third Affiliated Hospital of Guangzhou Medical University and was conducted in accordance with ethical standards.

## Author contributions

JL, XS, and YZ: Conceptualization and funding acquisition. JL: Formal analysis. JL and HL: Methodology. JL, HL, and DZ: Writing – original draft. HQ, XS, and YZ: Writing – review & editing. All authors contributed to the article and approved the submitted version.
